# Real-time PCR diagnosis of *Schistosoma japonicum* in low transmission areas of China

**DOI:** 10.1186/s40249-018-0390-y

**Published:** 2018-01-31

**Authors:** Pei He, Catherine A. Gordon, Gail M. Williams, Yuesheng Li, Yuanyuan Wang, Junjian Hu, Darren J. Gray, Allen G. Ross, Donald Harn, Donald P. McManus

**Affiliations:** 1 Hunan Institute of Parasitic Diseases, Yueyang, People’s Republic of China; 2 Molecular Parasitology Laboratory, Infectious Diseases Division, QIMR Berghofer Medical Research Institute, Brisbane, Australia; 3 Discipline of Epidemiology and Biostatistics, School of Population Health, University of Queensland, Brisbane, Australia; 4 Research School of Population Health, The Australian National University, Canberra, Australia; 5 Menzies Health Institute Queensland, Griffith University, Gold Coast, QLD Australia; 6 University of Georgia, College of Veterinary Medicine, Athens, GA USA

**Keywords:** Schistosomiasis, *Schistosoma Japonicum*, China, Real-time PCR, Miracidium hatching test, Kato-Katz, FEA-SD, Bovine, Human

## Abstract

**Background:**

Schistosomiasis in the People’s Republic of China (PRC) can be traced back to antiquity. In the past 60 years, the Chinese government has made great efforts to control this persistent disease with elimination slated by 2020 through the implementation of a comprehensive control strategy. This strategy aims to reduce the role of bovines and humans as sources of infection as a pre-requisite for elimination through transmission interruption. The goal of elimination will be achievable only by the implementation of a sustainable surveillance and control system, with sensitive diagnosis a key feature so that the true disease burden is not underestimated. Currently used diagnostics lack the necessary sensitivity to accurately determine the prevalence of *Schistosoma japonicum* infection in areas with low infection intensities. It is of critical importance to find and treat people and to identify animals with low-level infections if the National Control Programme for China is to achieve schistosomiasis elimination.

**Methods:**

We evaluated a real-time polymerase chain reaction (qPCR) assay using 633 human stool samples collected from five villages in Hunan, Anhui, Hubei, and Jiangxi provinces, and 182 bovine (70 cattle and 112 buffalo) stool samples obtained from four villages in Hunan, Anhui, and Jiangxi provinces in the PRC. All stool samples were subjected to the miracidium hatching test (MHT, a diagnostic procedure used in the National Schistosomiasis Control Programme) and the qPCR assay. Samples positive by MHT were subjected to either the Kato-Katz technique for humans, or the formalin-ethyl acetate sedimentation-digestion (FEA-SD) procedure for bovines, to determine infection intensities.

**Results:**

The qPCR assay exhibited a high level of sensitivity in the detection of *S. japonicum* infections. With both the human and bovine samples, a significantly higher prevalence was determined using the qPCR assay (11.06% humans, 24.73% bovines) than with the MHT (0.93% humans, 7.69% bovines). The animal contamination index (calculated using data obtained with the qPCR technique) for all positive bovines was 27 618 000 eggs per day, indicating a considerable amount of environmental egg contamination that would be underestimated using less sensitive diagnostic procedures.

**Conclusions:**

The qPCR assay we have evaluated will be applicable as a future field diagnostic and surveillance tool in low-transmission zones where schistosomiasis elimination is targeted and for monitoring post-intervention areas to verify that elimination has been maintained.

**Electronic supplementary material:**

The online version of this article (10.1186/s40249-018-0390-y) contains supplementary material, which is available to authorized users.

## Multilingual abstracts

Please see Additional file 1 for translation of the abstract into the six official working languages of the United Nations.

## Background

Schistosomiasis japonica, a zoonotic parasitic disease, remains of economic and public health concern in the People’s Republic of China (PRC), with 40 million people at risk of infection [1–3]. The government of PRC has been highly successful in reducing the prevalence of schistosomiasis in a number of endemic areas. As of 2013, the PRC had achieved transmission interruption in five out of 12 endemic provinces, and controlled transmission in four other endemic provinces now classified as low transmission areas (prevalence of heavy intensity infections < 5%) [4, 5]. We found a significant reduction in the number of cases, from an estimated 12 million in 1949 to an estimated 184 943 by the end of 2013 [4–6], so that now schistosomiasis elimination (prevalence < 1%) is a realistic and achievable goal for China. To certify elimination, and to guide control strategies at different thresholds of schistosomiasis transmission, accurate diagnostics, mathematical modelling, and rigorous surveillance methods are critical to estimate disease trends and to assess the effectiveness and impact of the control interventions/programs [7–10]. The zoonotic nature of Asian schistosomiasis complicates control efforts [11] and thus requires the development of additional tools for the control of animal hosts (particularly bovines) [12] and subsequent diagnosis/surveillance.

Highly sensitive and specific diagnostic tools, such as real-time PCR (qPCR), are required for effectively assessing the impact of control and elimination programs, and identifying at risk areas, disease reintroduction, or new transmission areas [8, 9, 13–15]. In areas of rebounding infections, newly infected areas, or after effective control measures have been put in place, the intensity of infection will be low and therefore likely to be missed by traditional microscopic techniques such as the Kato-Katz technique, the most commonly used for detecting schistosome eggs.

Reviews of the diagnostic armoury of techniques for schistosomiasis are available [16, 17]. Diagnostic procedures employed in the PRC include parasitological-based methods (miracidium hatching test [MHT], qualitative sedimentation, quantitative Kato-Katz), serological (antigen or antibody detection), histopathological (rectum biopsy), and molecular (polymerase chain reaction [PCR] and loop mediated isothermal amplification [LAMP]) methods [18–21]. The microscopy-based Kato-Katz technique recommended by the World Health Organization (WHO) to determine schistosome prevalence/intensity in humans [8] is the major tool used for stool examinations in the National Schistosomiasis Control Programme of the PRC. The main benefit of the Kato-Katz is its low cost (US$ 0.62/slide) [22–26] and ease of field application. However, its sensitivity is severely compromised in low-intensity infections and in areas of low prevalence [8]. For investigation of infection rates in animals (predominantly buffaloes and cattle but also sheep and goats) and humans, the MHT is used for the determination of infection status with faeces from positive samples subjected to sedimentation and microscopy to determine infection intensity [8, 27]. A recently developed procedure, the formalin-ethyl acetate sedimentation-digestion (FEA-SD) technique, has been shown to improve the visualisation of *Schistosoma japonicum* eggs in animal faeces [28]. Serological methods, particularly the indirect hemagglutination assay (IHA) and the enzyme-linked immunosorbent assay (ELISA) with soluble egg antigen, have also been used extensively [29, 30]. However, antibody-based serological methods can have low specificity due to cross-reactivity, and are generally unable to distinguish between current and past infections [31–33]. Nevertheless, there have been some important recent advances in immunological tests for schistosomiasis that detect anti-schistosome antibodies and/or circulating schistosome antigens mainly in plasma or serum [34, 35].

Conventional PCR (cPCR) and real-time PCR (qPCR) assays have been shown previously to be highly sensitive and specific for detection of schistosome eggs in human stool samples and could be a useful diagnostic in areas with low levels of schistosomiasis transmission [36–39]. Recently, qPCR-based tests have been used to diagnose schistosomiasis in humans and buffalo in the Philippines and the PRC [20, 22, 40–44]. In this study, we have compared a qPCR assay and the MHT for the diagnosis of *S. japonicum* in both humans and bovines from field-collected stool samples, to re-evaluate the prevalence of *S. japonicum* infections in low transmission areas of the PRC. Kato-Katz and FEA-SD on humans and bovines, respectively, were used to determine infection intensity on MHT-positive samples, and the results were directly compared with those obtained using the qPCR assay.

## Methods

### Ethics

Informed written consent was received from all human participants, and from animal owners in the study area. Ethical approval for both the human and animal work was provided by the Hunan Institute of Parasitic Diseases (HIPD) and QIMR Berghofer Medical Research Institute (QIMRB) Human Research Ethics Committee (P524) and QIMRB Animal Research Ethics Committee (P524). This study was performed in accordance with the recommendations of the Australian code of practice for the care and use of animals for scientific purposes, 2004.

### Study locations and sample collection

**Fig. 1:**
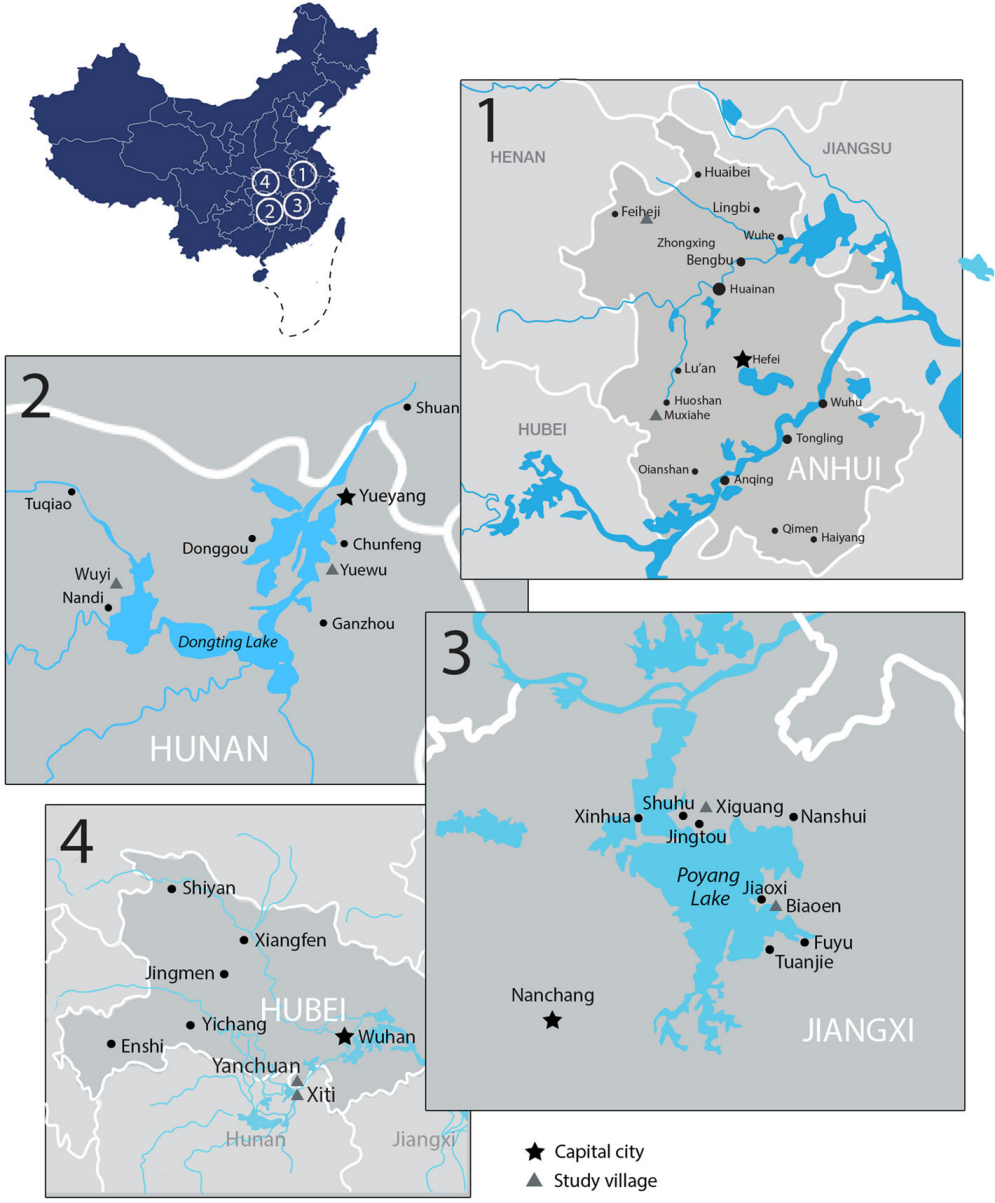
Map of the study area. 1: Zhongxing and Muxiahe villages, Anhui Province; 2: Yuewu and Wuyi villages, Hunan Province; 3: Xiguang and Biaoen villages, Jiangxi Province; 4: Xiti and Yanchuan villages, Hubei Province

This study was undertaken in three provinces in China in 2013. Human stool samples were collected from seven villages in Anhui, Hunan, Hubei, and Jiangxi provinces and bovine (from both cattle [*Bos* spp.] and buffalo [*Bubalus bubalis*]) stool samples were collected from four villages in Hunan and Jiangxi (Fig. 1, Table 1). All village residents enrolled in the study were 6–68 years of age. Hubei samples were subjected to qPCR only.

**Table 1:** Breakdown of stool samples by type and village

		Stool samples			
Province	Village	Humans	Bovines	Cattle	Buffalo
Anhui	Zhongxing	89	NA	NA	NA
	Muxiahe	69	NA	NA	NA
Jiangxi	Xiguang	107	63	56	7
	Biaoen	100	51	3	48
Hunan	Wuyi	NA	38	0	38
	Yuewu	63	30	11	19
Hubei	Yanchuan	82	NA	NA	NA
	Xiti	123	NA	NA	NA
	Total	633	182	70	112

*NA*: Not Applicable

**Fig. 2:**
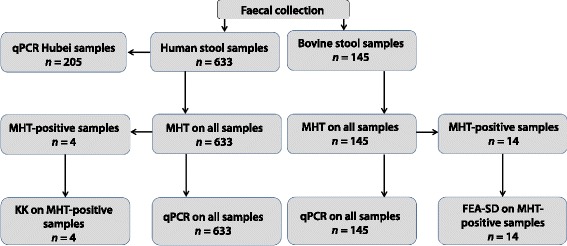
Flow diagram showing breakdown of the diagnostic techniques and numbers of human and bovine stool samples subjected to analysis

Human stools were collected and transferred on the same day to a local county anti-schistosomiasis station laboratory and examined using the MHT and Kato-Katz procedures [22, 28, 43]. Two stool samples were collected on different days from each participant. MHT was performed on both stool samples, and Kato-Katz (three slides per stool sample) carried out on MHT-positive samples only. Approximately 2 g of each of the first-day-only collected human stool were placed individually into a 5 ml tube, fixed with sufficient 100% ethanol to cover the sample, and transported to a laboratory at the HIPD for subsequent DNA extraction and qPCR analysis (Fig. 2).

Fresh faecal samples from bovines were collected intra-rectally from an individual animal (or a fresh, recently deposited sample) and placed into a labeled (with identity number, village and owner name) container. The bovine stool samples were subjected to the MHT and those positive were further examined by FEA-SD [22, 28, 43]. Prior to the MHT, 2 g of stool was removed for DNA extraction and qPCR analysis (Fig. 2).

### Miracidium hatching test

All human stool samples from Anhui, Jiangxi, and Hunan provinces, and all bovine stool samples were examined by the MHT using a previously published method [24]. In brief, the test involves concentration of eggs from faecal samples through a nylon tissue bag and their suspension in distilled water in a flask. Miracidia hatching from ova are visualized microscopically and their presence is an indication of infection. Flasks were checked for miracidium hatching at 4, 6, 8, and 24 h.

### Kato-Katz procedure

The Kato-Katz was performed as previously described by using nylon screens and a standard volume plastic template representing approximately 41.7 mg of stool [45]. Three slides were prepared from each homogenized stool sample, and examined under a light microscope by trained personnel. The Kato-Katz was done on MHT-positive samples only in order to obtain intensity of infection data.

### Formal-ethyl alcohol sedimentation technique

The FEA-SD was performed essentially as previously described [28] with the exception that the entire contents of only one (instead of two) of the final tubes was read by light microscopy. The FEA-SD was done on MHT-positive samples only, to calculate infection intensity.

### DNA extraction

DNA was extracted from 200 mg of human or bovine stool using the QIAamp DNA Stool Mini Kit (QIAGEN, Hilden, Germany) following procedures described in the company instruction manual. DNA concentrations and quality were determined using a NanoDrop Lite (Thermo Scientific, Waltham, USA).

### Real-time PCR

The qPCR assay was performed on all human and bovine samples. The qPCR targeted the NADH dehydrogenase I (*nad1*) mitochondrial gene as previously reported [20–22], with the inclusion of BSA to the qPCR reaction mix. Primer sequences were as follows, SjND1FW (forward: 5′-TGR TTT AGA TGA TTT GGG TGT GC-3′) and SjND1RV (reverse: 5´-AAC CCC CAC AGTCAC TAG CAT AA-3′) [40, 41]. Briefly, reaction mixtures of 20 μl were prepared containing 10 μl 2 × SYBR® Select Master Mix (ABI), 150 nmol/L of each primer, 2 μl of extracted DNA template and Ultra Pure BSA (Ambion, Austin, TX, USA) to a final concentration of 0.1 mg/ml. No-template controls (NTC) containing water instead of DNA template, and positive controls containing *S. japonicum* egg DNA as template were run with each assay. The qPCR was performed on a StepOnePlus™ Real-Time PCR System (Applied Biosystems, California, USA). The PCR cycling conditions were as follows: 2 min initialization at 50 °C, 10 min denaturation at 95 °C, followed by 40 cycles of 15 s denaturation at 95 °C, 60 s annealing at 60 °C, 90 s extension at 72 °C and a final dissociation phase at 60–95 °C. Melt curve analysis was performed for each PCR. The hybridisation temperature for the primer set was 66.25 °C. Efficacy for each PCR run was 1.00.

A standard curve was prepared to determine the equivalent egg number relating to cycle threshold (Ct) scores. Stool samples known to be negative for *S. japonicum* were seeded with 1000 eggs and DNA extracted. A serial 1:10 dilution series was made and run in triplicate. The results of this assay were then used as the standard curve.

### qPCR validation

A series of seeding experiments were performed using seeded eggs purified from the liver of an experimentally infected mouse to validate the qPCR assay. One, two and five eggs were seeded into 200 mg of known negative stool samples (10 stool repeats for each egg number) and the DNA extracted. These samples were then subjected to qPCR to evaluate the standard curve. Thirty human stool samples, as negative controls, were collected from subjects resident in a non-endemic schistosomiasis area. These stool samples were examined via light microscopy to confirm they were schistosome-egg-negative and subjected to the *S. japonicum*-specific qPCR assay.

### Statistical analysis

All results were stored in Microsoft Excel (2010) and Microsoft access databases (2010), and the data analyzed by SPSS 17.0 and SAS 9.3 (SAS Institute, Cary, NC). A sample was considered positive if at least one miracidium was observed by the MHT, or if the Ct score was < 35 by qPCR. The GMEPG (geometric mean eggs per gram of faeces) was calculated on positive faecal samples for the Kato-Katz, FEA-SD and qPCR using log-transformed egg counts. 95% confidence limits were calculated using the standard formulae based on the prevalence (binomial distribution) and the lognormal distribution (infection intensity). The relative diagnostic sensitivity and specificity of the MHT and qPCR was calculated using the combined results of both tests (excluding Hubei village results) as the reference standard. The sensitivity and specificity of the Kato-Katz and FEA-SD were not calculated as they were performed only on samples positive by MHT. *P*-values were calculated using McNemar’s test.

The animal contamination index (ACI) was derived using a previously published formula [46] using data obtained with the FEA-SD techniques and PCR assay.

ACI = [arithmetic mean epg (of infected bovines)] × [number of infected bovines] × [grams fecal weight].

We used 25 kg as a conservative estimate of the amount of stool excreted daily by buffalo and cattle, and 250 g for humans to calculate the ACI [47, 48].

## Results

### qPCR validation

Negative human stool samples seeded with a known number (1, 2, and 5) of *S. japonicum* eggs, were subjected to the qPCR assay. All samples (*n* = 30) were positive by qPCR, indicating the high sensitivity of the qPCR.

The specificity of the qPCR was determined using human stool samples collected from individuals resident in non-endemic areas. The stool samples were examined by light microscopy (Kato-Katz); hookworm, pinworm, and roundworm eggs were present but no eggs of *S. japonicum* were identified. All these control stool samples were also negative by the qPCR, reinforcing the specificity of the assay with no cross-reactivity evident with other commonly present parasitic helminths.

### Prevalence

In total, 633 human stool samples from seven villages and 182 bovine (70 cattle, 112 buffalo) stool samples collected from four villages were examined in this study (Fig. 2, Table 1). The prevalence of *S. japonicum* determined by qPCR in humans was 11.06%, while the prevalence based on MHT was significantly lower at 0.93% (Table 2). MHT found positive cases in only two out of five villages, while qPCR identified positive cases in all five villages. The prevalence determined by qPCR in each village ranged from 5.80% (95% *CI:* 1.42–11.45) in Muxiahe to 26.98% (95% *CI*: 15.72–38.25) in Yuewu (Table 2).

**Table 2:** Prevalence of *S. japonicum* in humans by the MHT and qPCR assay

			MHT			qPCR			
Province	Village	*N*	No. Positive	Prevalence (%)	95% *CI*	No. Positive	Prevalence (%)	95% *CI*	*P*-value (McNemar)
Anhui	Zhongxing	89	0	0.00	.	9	10.11	3.73, 16.50	0.003
	Muxiahe	69	0	0.00	.	4	5.80	1.42, 11.45	0.046
Jiangxi	Xiguang	107	1	0.93	−0.92, 2.79	11	10.28	4.43, 16.13	0.002
	Biaoen	100	0	0.00	.	13	13.00	6.29, 19.71	< 0.001
Hunan	Yuewu	63	3	4.76	−0.64, 10.17	17	26.98	15.72, 38.25	< 0.001
Hubei	Xiti	123	.	.	.	8	6.50	2.08, 10.92	0.0043
	Yanchuan	82	.	.	.	8	9.76	3.20, 16.32	0.004
	**Total**	**633**	**4**	**0.93**	**0.02, 1.85**	**70**	**11.06**	**9.46, 15.78**	**< 0.0001**

The final row of each table is in bold to denote that these are total numbers

The prevalence of schistosomiasis in bovines determined by qPCR was 24.73% and 7.69% by the MHT (Table 3). Village prevalence determined by qPCR ranged from 13.16% (95% *CI*: 1.90–24.42) in Wuyi to 56.67% (95% *CI*: 37.85–75.49) in Yuewu (Table 3).

**Table 3:** Prevalence of *S. japonicum* in bovines by the MHT and qPCR assay

		MHT			qPCR			
Village	*N*	No. positive	Prevalence (%)	95% *CI*	No. positive	Prevalence (%)	95% *CI*	*P*-value (McNemar)
Xiguang	63	5	7.90	1.07, 14.80	14	22.22	11.67, 32.78	0.007
Biaoen	51	1	1.96	−2.00, 5.90	9	17.65	6.82, 28.48	0.005
Wuyi	38	1	2.63	−2.70 7.96	5	13.16	1.90, 24.42	0.046
Yuewu	30	7	2.33	7.27, 39.40	17	56.67	37.85, 75.49	0.002
Animal type								
Cattle	70	9	12.90	4.82, 20.90	23	32.86	21.58, 44.14	0.0005
Buffalo	112	5	4.46	0.58, 8.34	22	19.64	12.17, 27.12	< 0.0001
Total	**182**	**14**	**7.69**	**3.78, 11.60**	**45**	**24.73**	**18.40, 31.05**	**< 0.0001**

The final row of each table is in bold to denote that these are total numbers

### Infection intensity

The intensity of *S. japonicum* infection, calculated as the GMEPG, obtained by qPCR was quantified by comparing Ct scores from unknown samples with the standard curve [22, 43, 44]. The GMEPG determined by qPCR for humans was 3.73, and 5.08 by Kato-Katz (Table 4).

**Table 4:** GMEPG of *S. japonicum* in humans by Kato-Katz and qPCR assay

Village	Kato-Katz^a^			qPCR		
	No. MHT positive	GMEPG	95% *CI*	No. positive	GMEPG	95% *CI*
Zhongxing	0	.	.	9	3.24	2.61, 4.03
Muxiahe	0	.	.	4	2.16	0.99, 4.70
Xiguang	1	12.04	.	11	7.00	3.36, 14.59
Biaoen	0	.	.	13	3.48	2.70, 4.49
Yuewu	3	3.81	2.15, 3.36	17	4.85	3.36, 7.00
Xiti	.	.	.	8	2.20	1.50, 3.24
Yanchuan	.	.	.	8	2.54	1.73, 3.72
Total	**4**	**5.08**	**2.27, 14.35**	**70**	**3.73**	**3.13, 4.45**

^a^Kato-Katz was only performed on MHT-positive samples

The final row of each table is in bold to denote that these are total numbers

The GMEPG of bovine samples by qPCR was 7.74 (95% *CI:* 7.57–7.91) and 1.30 (95% *CI*: 0.99–1.59) by FEA-SD (Table 5) (*P* ≤ 0.00001).

**Table 5:** Intensity of *S. japonicum* infection (GMEPG) in cattle and buffalo by village and bovine type (cattle or buffalo)

	MHT	FEA-SD			qPCR		
Village	No. MHT positive^a^	No. positive	GMEPG	95% *CI*	No. positive	GMEPG	95% *CI*
Xiguang	5	4	0.38	0.06, 2.42	14	7.18	2.75, 18.76
Biaoen	1	0	.	.	9	3.18	2.13, 4.76
Wuyi	1	1	1.80	.	5	15.55	1.93, 125.28
Yuewu	7	7	2.43	1.16, 5.10	17	10.73	5.88, 19.56
Bovine type							
Cattle	9	7	0.87	0.39, 1.34	23	7.67	4.06, 14.49
Buffalo	5	5	2.29	2.18, 2.42	22	7.81	4.40, 13.86
Total	**14**	**12**	**1.30**	**0.99, 1.59**	**45**	**7.74**	**7.57, 7.91**

^a^The FEA-SD was performed on MHT-positive samples only; two MHT-positive samples were negative by FEA-SD

The final row of each table is in bold to denote that these are total numbers

The ACI calculated for the cattle and buffalo combined was 27 608 000 eggs per day by the qPCR assay and 672 000 eggs per day by FEA-SD (Table 6). Based on these results the percentage environmental contamination due to cattle and buffalo was calculated using the FEA-SD and qPCR data. Using the FEA-SD and qPCR values, cattle were shown responsible, respectively, for 28.81% and 38.80% of environmental contamination. For buffalo the environmental contamination was 71.19% by FEA-SD and 61.20% by qPCR (Table 6).

**Table 6:** Animal contamination index (ACI)

		No. positive	Arithmetic mean EPG in positives	ACI per infected bovine or human per day	ACI all infected bovines or human per day
Cattle	FEA-SD^a^	7	2.04	51 000	357 000
	qPCR	23	27.38	684 500	15 743 500
Water buffalo	FEA-SD^a^	5	2.52	63 000	315 000
	qPCR	22	21.59	539 750	11 874 500
Bovine	FEA-SD^a^	12	2.24	56 000	672 000
	qPCR	45	24.55	613 733.3	27 618 000
Human	Kato-Katz	4	12.22	3055	12 220
	qPCR	70	6.74	1685	117 950

^a^The FEA-SD was performed on MHT-positive samples only; two MHT-positive samples were negative by FEA-SD

### Sensitivity and specificity of the MHT and qPCR

The sensitivity and specificity of the MHT and qPCR were calculated using the results of both techniques as the reference standard. For humans, the specificity of the qPCR assay was 100% and sensitivity was 100%, while for the MHT specificity was 100% and sensitivity was 7.40%. For bovines, the sensitivity and specificity of the qPCR assay were 96.83% and 100%, respectively, and the sensitivity and specificity of the MHT were 30.43% and 100%, respectively.

## Discussion

The prevalence of *S. japonicum* in the PRC is at the lowest recorded level since large-scale control programs were rolled out from the 1950’s. As of 2013, 296 of 454 endemic counties had reached the status of transmission interruption [4]. Transmission interruption is defined as: no locally acquired schistosomiasis in humans and domestic animals for five years; and no *Oncomelania* spp. snails found in careful surveys for two years [49].

In the PRC, field diagnosis for schistosomiasis relies on indirect haemagglutination (IHA) serology and detection of parasite eggs in stool using the MHT or Kato-Katz [50]. These tools are generally easy to implement, inexpensive, provide rapid results, and are therefore widely used. However, whereas IHA serology is highly sensitive, it can yield false-positive results after curative praziquantel treatment due to the inability of the test to differentiate between past and current infections, as well as displaying cross-reactivity with other parasitic infections [31–33, 51, 52]. Kato-Katz is considered to be the gold standard for the diagnosis of schistosomiasis, but lacks sensitivity in low and mid intensity infections [53–56]. While the MHT is greatly affected by freshness of stool, the temperature of the environment and the pH of the water used in the test. Therefore, the MHT and Kato-Katz may result in false negatives and the missed cases can then become a transmission source of *S. japonicum* [57]. Schistosomiasis is a chronic infection and intensity of infection or faecal egg output does not always correlate with disease intensity [58]. Most individuals in a schistosome-endemic population will have low infection levels which are often deemed less important as they are not associated with severe pathology; however, the morbidity associated with schistosome infections, in people with light infection intensities, tends to result in reduced productivity due to a reduced ability to work and concentrate at school, abdominal pain, growth retardation, exercise intolerance, lower work capacity, diarrhoea, anaemia and malnutrition, which has an overall economic loss for the country [59–61].

It is of considerable epidemiological importance to find and treat people with low-level infections if elimination programs are to be effective and sustained. Molecular diagnostics are sensitive and specific with qPCR-based assays already having been proven for identifying schistosome infections in humans and animals [22, 37, 38, 41, 43, 62, 63]. Real-time PCR is rapid, sensitive and reproducible, and does not require post amplification processing, such as gel electrophoresis which is required for conventional PCR. qPCR is semi-quantitative and so the intensity of infection can be calculated from Ct scores [40, 41, 44].

Prevalence of schistosomiasis in humans from three villages (Zhongxing and Muxiahe in Anhui Province, and Biaoen in Jiangxi Province) by the MHT was 0%, while in the other two villages it ranged from 0.93–4.76%. By qPCR, prevalence in MHT-negative villages ranged from 5.80–13.00%, highlighting the lack of sensitivity of the MHT (Table 2). Sensitivity of the MHT in humans was 7.40% and in bovines 30.43%, compared with 100% and 97.83%, respectively, for the qPCR. Previous studies have indicated the FEA-SD method has a similar level of sensitivity to the qPCR assay [22, 43], but in this study the FEA-SD was used only to provide intensity of infection data for MHT-positive samples due to the laborious nature of the technique [28, 43]. Similarly, Kato-Katz was only performed on human stool samples positive by MHT.

The highest prevalence for both humans and bovines was obtained with the qPCR assay (11.06% humans, 24.73% bovines) while the MHT gave the lowest prevalence for humans (0.93% MHT) and bovines (7.69% MHT) (Table 2, Table 3). Therefore, due to its higher sensitivity, the qPCR was able to identify considerably more positive cases than the Kato-Katz. Hunan Province had the highest prevalence by qPCR (26.98%) followed by Jiangxi (11.59%), Anhui (8.23%), and Hubei (7.80%). In comparison the MHT found very few cases (0% in three of the five villages where it was utilized), thereby underestimating the true prevalence of schistosomiasis in endemic provinces with implications for control.

Overall, a significantly higher (*P* <  0.0001) GMEPG was obtained with the qPCR assay than the FEA-SD for bovines. This is likely due to the higher sensitivity of the qPCR which was able to identify more infected animals. Due to the low sensitivity of the MHT it would be expected that these samples would have a higher EPG. Two samples positive by MHT were negative by FEA-SD and were thus unable to be quantified. By the qPCR these samples had an EPG of close to 1. As the qPCR is a more sensitive technique, it was able to identify light infections missed by the MHT. However, one human sample, with an estimated arithmetic EPG of 168 by qPCR, was negative by the MHT. This may have been due either to the fact the sample was not sufficiently fresh, or the conditions were sub-optimal (e.g. low temperature) at the time when the test was undertaken. Similarly, one bovine samples was negative by qPCR and FEA-SD, but positive by MHT, and another was positive by both FEA-SD and MHT, but negative by qPCR. This may have been due to the much larger stool size used for the MHT and FEA-SD compared with the qPCR assay. With the MHT, 150 g of stool was processed and 10 g for the FEA-SD, whereas only 200 mg of stool was used for DNA extraction and the subsequent qPCR assay. The FEA-SD was only performed on MHT-positive samples which may have affected its diagnostic effectiveness.

The ACI was calculated using the FEA-SD, Kato-Katz, and qPCR assay data. The ACI for individual bovines was higher using data from the qPCR assay compared with the FEA-SD method which translated to a much higher total environmental contamination with the former procedure. This was both due to the higher EPG calculated by the qPCR assay, and the larger number of samples that were positive by the qPCR than by the MHT. The FEA-SD was only performed on MHT-positive bovines for intensity of infection calculations. There was a difference in the methods of 26 946 000 eggs between the total ACI for all bovines, representing a considerable amount of environmental schistosome egg contamination that would be missed when using the MHT. Similarly for the human stool samples, the total ACI per individual was considerably higher by the qPCR than by the Kato-Katz even though the latter had a higher EPG, representing an additional 105 000 eggs contaminating the environment missed by Kato-Katz. As with the bovines, the MHT was performed on all human stool samples and the Kato-Katz performed on those that were positive by MHT for intensity calculations. The MHT was likely only picking up higher intensity infections (*n* = 4) resulting in a higher EPG. As the qPCR picked up significantly more positive infections (*n* = 70), the total ACI determined using data obtained with the qPCR assay was much higher. The environmental contamination, measured by the total ACI, was much higher for bovines than humans, but the ACI calculated using the qPCR data indicates that humans can also contribute to transmission. The ACI was higher in cattle than buffalo as the former have been shown to be more susceptible to infection with *S. japonicum* [22, 43, 64, 65].

The relatively high prevalence of *S. japonicum* determined for humans and bovines in this study by qPCR, and the high individual ACI calculated for bovines using the qPCR and FEA-SD data, indicate that schistosomiasis still poses a public health threat in some areas of the PRC. The low GMEPG emphasizes the importance of using more sensitive detection methods than which are currently used for surveillance in the PRC. Undetected cases can cause rebound infections in areas thought to be controlled [66], as occurred in eight counties in Sichuan Province that were characterized as either “transmission control” [7] or “transmission interruption” [67] but showed re-emergence with an average “return time” of 8 years following the cessation of active control interventions [68].

While the qPCR assay (US$ 9.20 per sample) is a highly sensitive technique, it is relatively expensive, particularly when compared with the Kato-Katz method (US$ 0.62) [22]. It is, therefore, unlikely to be implemented as a large scale diagnostic tool until the costs for the assay are markedly reduced which will inevitably happen in time. However, it could be used quite effectively as a surveillance tool on a subset of the population in an endemic area and, in that way, measure the impact of implemented control strategies including assessing potential elimination [44]. Whilst cost-benefit analyses would be required, the outlay of cost on a highly sensitive diagnostic as schistosomiasis elimination approaches may well be cheaper in the long-term than the cost of re-emergence. Results from qPCR surveillance can also be used to create more accurate risk maps and disease modeling scenarios.

## Conclusions

The central government of the PRC has championed the target of achieving the elimination (i.e. reducing a locally acquired infection rate to zero) of schistosomiasis by 2025 through the implementation of a comprehensive control strategy which aims to eliminate the role of bovines and humans as the sources of infection for intermediate host snails as a pre-requisite for transmission interruption. This goal will be achievable only by the formulation of a sustainable surveillance and control system, with highly sensitive diagnostics being the key. Complemented by a recently developed LAMP method for identifying infected *Oncomelania hupensis* snails [18, 69], the qPCR method we describe provides an additional tool for field diagnosis and schistosomiasis surveillance as the PRC’s slated intention to eliminate schistosomiasis becomes a reality.

## Additional file

Additional file 1: Multilingual abstracts in the six official working languages of the United Nations. (PDF 784 kb)

## Abbreviations

ACI: Animal contamination index
Ct: Cycle threshold
EPG: Egg per gram of faeces
FEA-SD: Formalin-ethyl acetate sedimentation-digestion
GMEPG: Geometric mean eggs per gram of faeces
HIPD: Hunan Institute of Parasitic Diseases
IHA: Indirect hemagglutination assay
LAMP: Loop mediated isothermal amplification
MHT: Miracidium hatching technique
*nad1*: NADH dehydrogenase I
NTC: No-template controls
PRC: People’s Republic of China
QIMRB: QIMR Berghofer Medical Research Institute
qPCR: Real-time polymerase chain reaction
WHO: World Health Organization
