# Supplementary material for: Real-time PCR diagnosis of Schistosoma japonicum in low transmission areas of China
Source: Infect Dis Poverty. 2018 Jan 31;7:8. doi: 10.1186/s40249-018-0390-y (PMC5796516; doi:10.1186/s40249-018-0390-y)
Supplement: Additional file 1: — Multilingual abstracts in the six official working languages of the United Nations. (PDF 784 kb) [file 40249_2018_390_MOESM1_ESM.pdf]

Translation of the abstract into the six official working languages of the United Nations

تشخيص البلهارسيا اليابانية بالزمن الحقيقي عن طريق تفاعل البوليميراز المتكرر في مناطق الانتشار المنخفضة في الصين

بي هي، كاثرين أ. غوردن، غيل م. ويليامز، لي يوشينغ، يوان يوان وانغ، جونجيان هو، دارين ج. غري، آلن ج. روس، دونالد هارن، دونالد ب. ماكمانوس

ملخص

خلفية البحث: يمكن ملاحظة أثر داء البلهارسيات في جمهورية الصين الشعبية منذ القِدم. في الستين سنةً الماضية، قامت الحكومة الصينية بجهودٍ كبيرة للتحكم بهذا المرض المستمر عن طريق القضاء عليه المتوقع بحلول عام 2020 وذلك بتنفيذ استراتيجية تحكمٍ شاملة. تهدف هذه الاستراتيجية إلى تخفيض دور المواشي والبشر كونهم مصدراً للعدوى كمطلب مسبَق للتمكن من القضاء عليها عن طريق قطع الانتشار. يتحقق ذلك الهدف فقط بتنفيذ نظام مراقبة وتحكم دائمين، مع وجود ميزة أساسية للتشخيص الحساس حتى لا يتم التقليل من شأن العبء الحقيقي لهذا المرض. إن علوم التشخيص المستخدمة حالياً ينقصها الحساسية المطلوبة لتحديد انتشار عدوى البلهارسيا اليابانية بدقة في المناطق ذات الشدات المنخفضة للعدوى. من المهم جداً إيجاد الأشخاص ومعالجتهم، كذلك تحديد الحيوانات ذات الإصابة الخفيفة إذا كان برنامج التحكم الوطني في الصين معنياً بالوصول إلى القضاء على داء المنشقات.

الطرق المستخدمة: قمنا بتقييم فحص تفاعل البوليميراز المتكرر بالزمن الحقيقي (qPCR) باستخدام 633 عينات من براز الإنسان تم جمعها من 5 قرى في مقاطعات هونان، أنهوي، هوبي وجيانجي، بالإضافة إلى 182 عينة من براز المواشي (70 بقرة و112 جاموساً) تم الحصول عليها من 4 قرى في مقاطعات هونان، أنهوي وجيانجي في جمهورية الصبن الشعبية. تم تعريض جميع عينات البراز إلى اختبار التفقيس (MHT) (وهو إجراء تشخيصي يستخدم في البرنامج الوطني للتحكم بداء البلهارسيات) كما خضعت لفحص تفاعل البوليميراز المتكرر. بالنسبة للعينات التي أظهرت نتيجةً إيجابية من اختبار MHT فقد خضعت بدورها إلى تقنية Kato-Katz للإنسان، وللمواشي خضعت لإجراء هضم الرواسب بخلات الايتيل فورمالين (FEA-SD)، وذلك لتحديد شدات العدوى.

النتائج: أظهر فحص تفاعل البوليميراز المتكرر درجةً عاليةً من الحساسية في كشف البلهارسيا اليابانية. في كل من العينات البشرية والحيوانية، تمت ملاحظة انتشار أعلى بشكل كبير باستخدام فحص qPCR (11.06% للبشر، 24.73% للمواشي) وذلك أعلى من نتائج اختبار MHT حيث كانت (0.93% للبشر، 7.69% للمواشي). بلغ مؤشر التلوث الحيواني (المحسوب باستخدام البيانات المستنبطة من تقنية qPRC) لكل المواشي الإيجابية 27,618,000 بيضة في اليوم الواحد، مما يشير إلى كمية هائلة من التلوث البيئي بالبيوض والذي يمكن أن تقل أهميته باستخدام إجراءات تشخيصية أقل حساسية.

الخلاصة: سيكون فحص تفاعل البوليميراز المتكرر qPRC الذي قمنا بتقييمه قابلاً للتطبيق كأداة مستقبلية للتشخيص والمراقبة في المناطق ذات الانتشار المنخفض حيث يكون القضاء على البلهارسيا هدفاً واضحاً، وفي المناطق التي تمر بمرحلة ما بعد الإصابة من أجل المراقبة وضمان سير عملية القضاء على المرض.

Translated from English version into Arabic by AyaAj, through

TRANSLATORS
WITHOUT BORDERS

在中国血吸虫病低度流行区采用实时荧光定量 PCR 检测日本血吸虫

Pei He, Catherine A. Gordon, Gail M. Williams, Li Yuesheng, Yuanyuan Wang, Junjian Hu, Darren J. Gray, Allen G. Ross, Donald Harn, Donald P. McManus

摘要

引言：中国血吸虫病的流行历史悠久。过去的 60 年里，中国政府在实施全面综合防治措施方面做出了巨大的努力，并旨在 2020 年实现消除血吸虫病。该措施主要目的是为了减少牛和人作为感染源的作用，这是通过传播阻断消除血吸虫病的先决条件，而只有应用敏感的检测方法才能使疫情不被低估。在有效的防治和监测措施下，消除血吸虫病的目标是可以达到的，但目前所用的诊断方法敏感性不够，难以在低度流行区检测到血吸虫感染。中国要实现消除血吸虫病的宏伟目标，研发一种能检测低水平感染的方法是至关重要的。

方法：用实时荧光定量核酸扩增检测系统(qPCR)和尼龙绢集卵孵化法(MHT)对湖南、安徽、湖北、江西 4 省 5 个村的 633 人和 182 头牛(70 头黄牛、112 头水牛)粪便样本进行检测。MHT 阳性的人粪和牛粪分别用加藤厚涂片法(Kato-Katz)和醋酸甲醛-乙酸乙酯沉淀消化法(FEA-SD)检测确定感染度。

结果：qPCR 检测日本血吸虫感染具有高敏感性。在人粪和牛粪样本检测中，qPCR 的血吸虫感染检测率(人粪 11.06%，牛粪 24.73%)高于 MHT(人粪 0.93%，牛粪 7.69%)。用 qPCR 检测阳性牛的污染指数为每天 27,618,000 个虫卵。结果表明，如用低敏感性的检测方法，大量虫卵污染环境的情况则会被低估。

结论：本研究报告的的 qPCR 法可作为今后在血吸虫病低度流行区现场诊断和监测的有效工具，并可用于监测消除血吸虫病后的疫情是否巩固。

Translated from English version into Chinese by Pei He

Diagnostique en temps réel en République Populaire de Chine du Schistosome japonicum dans les zones de faibles transmissions de Chine

Pei He, Catherine A. Gordon, Gail M. Williams, Li Yuesheng, Yuanyuan Wang, Junjian Hu, Darren J. Gray, Allen G. Ross, Donald Harn, Donald P. McManus

Résumé

Contexte: Le Schistosomiasis dans la République Populaire de Chine (RPC) remonte à l'antiquité Lors des 60 dernières années, le gouvernement chinois a fait de gros efforts pour contrôler cette maladie persistante en ciblant son éradication pour 2020 grâce à la mise en place d’une stratégie de contrôle complète. Cette stratégie a pour but de réduire le rôle de bovins et des humains comme les sources d'infection comme pré requis pour l'élimination par l'interruption de transmission. Le but de l'élimination sera réalisable seulement par la mise en œuvre d'une surveillance durable et d'un système de contrôle, avec le diagnostic sensible comme caractéristique clé afin que le vrai fardeau de la maladie ne soit pas sous-estimé. Le diagnostic actuellement utilisé manque la sensibilité nécessaire pour précisément déterminer la fréquence de l’infection Schistosoma japonicum dans des zones à intensités d'infection basses. Il est nécessaire de trouver et soigner les personnes et d’identifier les animaux à bas taux d’infection si le programme de contrôle national de Chine souhaite éradiquer le schistosomasis.

Méthodes: Nous avons évalué un essai de réaction en chaîne de polymères en temps réel (qPCR) utilisant 633 échantillons de selles humaines issus de cinq villages de Hunan, Anhui, Hubei et des provinces de Jiangxi et des échantillons de selles de 182 bovins (70 bétails et 112 buffles) obtenus de quatre villages dans Hunan, Anhui et des provinces de Jiangxi en RPC. Tous les échantillons de selles ont subi le test de hachures au miracidium (THM) (une procédure de diagnostique utilisée dans le programme de contrôle national du schistosomiasis) et l ‘essai qPCR. Les échantillons positifs au THM ont été soumis soit à la technique Kato-Katz pour les humains, ou à la procédure de digestion-sédimentation acétate éthylo-formol (DS-AEF) pour les bovins, afin de déterminer les intensités d’infection.

Résultats: L’essai qPCR a montré un fort taux de sensibilité dans la détection des infections de S. japonicum. Avec les échantillons humains et bovins, une prévalence plus haute significative a été déterminée en utilisant l’essai qPCR (11,06% humains, 24,73% bovins) qu’avec le THM L’index de contamination animal (calculé en utilisant les données obtenues avec la technique qPCR) pour tous les bovins positifs était 27 618 000 œufs par jour, ce qui indique un taux considérable de contamination d’oeuf environnemental qui serait sous-estimé dans le cas d’utilisation de procédures de diagnostique moins sensibles.

Conclusions: L’essai qPCR que nous avons évalué sera applicable comme diagnostique de terrain futur et outil de surveillance dans les zones de faible transmission où l’élimination du schistosomiasis est visée et afin de contrôler les zones post-intervention pour vérifier que l’élimination est toujours en vigueur.

Translated from English version into French by Emilie Rigault Fourcadier, through

TRANSLATORS WITHOUT BORDERS

Диагностика шистосомы японской при помощи методики полимеразной цепной реакции (ПЦР), проводимая в режиме реального времени в районах низкой плотности инфецирования Китая

Пэй Хэ, Катрин А. Гордон, Гэйл М. Уиллиамс, Ли Юэшэн, Юаньян Ван, Цзунцзянь Ху, Даррен Дж. Грэй, Аллен Г. Росс, Дональд Харн, Дональд П. МакМанус

Аннотация

Справочная информация: В Китайской Народной Республике (КНР) история шистосомоза восходит ко временам античности. За последние 60 лет, правительством Китая прилагаются значительные усилия с целью контроля этого устойчивого заболевания и полного его устранения к 2020 году посредством внедрения стратегии всеобъемлющего контроля. Указанная стратегия нацелена на снижение роли бычьих и людей в качестве источника инфецирования, необходимой состявляющей в этом случае является прерывание процесса инфецирования. Данная цель достижима

только при условии внедрения системы устойчивого наблюдения и контроля, включающей чувствительную диагностику в качестве ключевого параметра во избежание недооценки истиного уровня бремени болезни. Для точного определения распространённости инфекции *шистосомы японской (Schistosoma japonicum)* в районах с низкой плотностью интенсивности инфецирования используемой в настоящее время диагностике недостаёт требуемой чувствительности. Для успеха в достижении цели устранения шистомоза в рамках государственной программы контроля Китая чрезвычайно важно выявить и вылечить людей, а также выявить животных с низким уровнем инфецирования.
Методы: С использованием методики ПЦР в режиме реального времени (qPCR) мы произвели анализ проб, взятых из образцов кала 633 людей, проживающих в пяти деревнях провинций Хунань, Аньхой, Хубэй и Цзянси, а также из образцов кала 182 бычьих (70 коров/быков и 112 буйволов) из четырёх деревень провинций Хунаня, Аньхоя и Цзянси в КНР. По всем образцам кала были проведёны тест на вылупление мирацидия (МНТ) (процедура диагнстики, применяемая в рамках Программы по контролю шистосомоза), а также анализ проб по методике qPCR. С целью определения интенсивности инфецирования по случаям, давшим положительные результаты при анализе МНТ, был проведён либо анализ методом Като для людей, либо процедура седиментации-усвоения формалин-этилацетата（FEA-SD) для бычьих.
Результаты: Анализ проб по методу qPCR продемонстрировал высокй уровень чевствительности при выявлении инфекций *шистосомы японской*. В случаях с человеческими и бычьими образцами значительно более высокая распространённость заболевания была выявлена анализом проб по методу qPCR (11,06% у людей; 24,73% у бычьих), чем по методу МНТ (0,93% у людей; 7,69% у бычьих). Индекс заражаемости животных (рассчитываемый с использованием данных, полученных по методу qPCR) для всех бычьих с положительными результатами анализа составил 27 618 000 яиц в день, что указывает на значительный уровень зараженности яйцами окружающей среды. Данный параметр был бы недооценен при задействии менее чувствительного метода диагностики.
Выводы: Выполненный анализ проб по методу qPCR будет использован в будущем в качестве инструмента диагностики и наблюдения в зонах низкой плотности инфецирования, в которых поставлена цель устранения шистосомоза, а также для наблюдения в районах прошлого вмешательства с целью подтверждения факта полной ликвидации и контроля за ситуацией.

Translated from English version into Russian by Liudmila Tomanek (nee Volynets), through

TRANSLATORS WITHOUT BORDERS

Diagnóstico en tiempo real de Schistosoma japonicum mediante la técnica de PCR en áreas de baja transmisión en China

Pei He, Catherine A. Gordon, Gail M. Williams, Li Yuesheng, Yuanyuan Wang, Junjian Hu, Darren J. Gray, Allen G. Ross, Donald Harn, Donald P. McManus

Resumen
Contexto: El origen de la esquistosomiasis en la República Popular China (RPC) se remonta a la antigüedad. Durante los últimos 60 años, el gobierno chino ha hecho un gran esfuerzo para controlar esta persistente enfermedad. Su erradicación está estimada para el 2020, gracias a la adopción de una estrategia de control global que pretende reducir el papel de las reses bovinas y los humanos como fuentes de infección como prerrequisito para la erradicación de la enfermedad, al interrumpir el contagio. El objetivo de erradicación será posible solo mediante de la implementación de un sistema de supervivencia y control sostenible, haciendo del diagnóstico sensible la clave, de modo que no se subestime la verdadera carga de morbidad de la enfermedad. Los diagnósticos actuales carecen de la sensibilidad necesaria para determinar con exactitud la prevalencia de la infección por *Schistosoma japonicum* en zonas con un bajo índice de infección. Es de vital importancia hallar y tratar a las personas e identificar a los animales con infecciones de bajo nivel si el Programa Nacional de Control de China logra erradicar la esquistosomiasis.
Métodos: Evaluación en tiempo real de un ensayo de reacción en cadena de la polimerasa (PCR, por sus siglas en inglés) haciendo uso de muestras de 633 heces humanas procedentes de habitantes de cinco pueblos de las provincias de Hunan, Anhui, Hubei, and Jiangxi, además de 182 muestras de heces bovinas (70 reses vacunas y 112 búfalos) ubicadas en cuatro pueblos de las provincias de Hunan, Anhui, and Jiangxi, en la RPC. Todas ellas estuvieron sujetas a la prueba de incubación de miracidia, un tipo de procedimiento de diagnóstico utilizado en el Programa de Nacional de Control de Esquistosomiasis, y al ensayo PCR. Las

muestras positivas en la prueba de incubación de miracidia fueron examinadas mediante el método de Kato-Katz en el caso de los humanos, o la técnica de sedimentación-digestión con formol-acetato de etilo（F.A.E.）en el caso de los animales, para determinar la intensidad de la infección.
Resultados: El ensayo PCR mostró un alto nivel de sensibilidad en la detección de infecciones por *S. japonicum*. Tanto en las muestras humanas como en las bovinas se determinó una prevalencia mucho mayor mediante el ensayo PCR (11,06% de humanos, 24,73% de bovinos) que con la prueba de incubación de miracidia (0,93% de humanos, 7,69% de bovinos). El índice de contaminación animal (calculado mediante los datos obtenidos con la técnica PCR) para todos los positivos bovinos fue de 27 618 000 huevos por día, lo que constituye una cantidad considerable de contaminación ambiental de huevos, la cual se subestimaría haciendo uso de procedimientos de diagnóstico menos sensibles.
Conclusiones: El ensayo PCR evaluado será aplicable como campo de diagnóstico en un futuro, así como herramienta de supervivencia en áreas de baja transmisión en las que se tiene como objetivo la erradicación de la esquistosomiasis, además de para llevar el control tras realizarse la intervención, pudiendo verificarse de este modo que la erradicación continúa siendo efectiva.

Translated from English version into Spanish by Maria_v95, through

TRANSLATORS
WITHOUT BORDERS
